# Global patterns of sequence evolution in *Drosophila*

**DOI:** 10.1186/1471-2164-8-408

**Published:** 2007-11-09

**Authors:** Miguel Gallach, Vicente Arnau, Ignacio Marín

**Affiliations:** 1 Departamento de Genética. Universidad de Valencia. Valencia, Spain; 2 Departamento de Informática. Universidad de Valencia. Valencia, Spain; 3 Instituto de Biomedicina de Valencia. Consejo Superior de Investigaciones Científicas (IBV-CSIC). Valencia, Spain

## Abstract

**Background:**

Sequencing of the genomes of several *Drosophila *allows for the first precise analyses of how global sequence patterns change among multiple, closely related animal species. A basic question is whether there are characteristic features that differentiate chromosomes within a species or between different species.

**Results:**

We explored the euchromatin of the chromosomes of seven *Drosophila *species to establish their global patterns of DNA sequence diversity. Between species, differences in the types and amounts of simple sequence repeats were found. Within each species, the autosomes have almost identical oligonucleotide profiles. However, X chromosomes and autosomes have, in all species, a qualitatively different composition. The X chromosomes are less complex than the autosomes, containing both a higher amount of simple DNA sequences and, in several cases, chromosome-specific repetitive sequences. Moreover, we show that the right arm of the X chromosome of *Drosophila pseudoobscura*, which evolved from an autosome 10 – 18 millions of years ago, has a composition which is identical to that of the original, left arm of the X chromosome.

**Conclusion:**

The consistent differences among species, differences among X chromosomes and autosomes and the convergent evolution of X and neo-X chromosomes demonstrate that strong forces are acting on drosophilid genomes to generate peculiar chromosomal landscapes. We discuss the relationships of the patterns observed with differential recombination and mutation rates and with the process of dosage compensation.

## Background

*Drosophila melanogaster *has been one of the most important animal models since the beginning of modern genetics. It was therefore obvious that its genome should be one of the first to be sequenced. Genome projects of eleven other *Drosophila *species are now almost finished and this provides the first opportunity to establish the global patterns of short-term genome evolution, at both the genic and chromosomal levels, in metazoans. These data are contributing to a detailed view of gene evolution, intron length evolution, selective constraints acting on non-coding sequences and many other processes [1-6].

A classical problem in molecular genetics and evolution is the characterization of the complex relationships that exist between coding sequences and repetitive elements. Changes in the repetitive component of a genome may influence all kinds of significant phenomena, from gene expression to genome size. Studies comparing *Drosophila *species were among the first that demonstrated that both the satellites and the middle repetitive component, mostly mobile elements, of closely related species may be very different (summarized in [7]). The available genomic information for drosophilids may now contribute to obtain a much more detailed picture of the impact of repetitive sequences on genome and species evolution. One of the most interesting aspects to explore is the effect that changes in repetitive DNA content may have on chromosome structure and function. Until recently, this type of study was based on conventional cytogenetic analyses plus *in situ *hybridization with repetitive probes. The presence in drosophilids of high-quality polytene chromosomes allowed to obtain some of the most significant results of the pre-genomic era. These included the localization of mobile elements and the determination of their rates of transposition (reviewed in [8]), the localization of satellite sequences, including some specifically dispersed along the euchromatin of the X chromosome [9,10] or the demonstration that several simple DNA repeats were preferentially concentrated on the euchromatin of some chromosomes [11-13]. Particularly, Lowenhaupt *et al*. [13] used *in situ *hybridization with mono- and dinucleotides to obtain four main conclusions. First, they found that Drosophila subgenus species had consistently more repeats than species of the Sophophora subgenus. Later, this difference between subgenera was indirectly confirmed [14-17]. Second, they detected that three types of simple DNA repeats (CA/TG, CT/AG and C/G) were more abundant on the X chromosomes than on the autosomes of all the examined species, a result also later confirmed using different approaches [18-20]. Their third conclusion was that a chromosomal arm in *D. pseudoobscura *and *D. miranda*, which was involved in a translocation with the X chromosome and thus became a second X chromosomal arm (named XR), was also enriched for these repeats. Finally, they found that the X_2 _chromosome of *D. miranda *– originally an autosome but that now segregates as a second X chromosome because its homolog, called neo-Y, is attached in this species to the Y chromosome – contained also some regions with repeat enrichment. In *Drosophila*, and due to the fact that the Y chromosome lacks most of the genes present on the X, the single X chromosome of males is hypertranscribed to generate roughly the same amount of products that the two X chromosomes of the females, a process known as dosage compensation (reviewed in [21,22]). The fact that the homolog of the XR arm of *D. pseudoobscura *and *D. miranda *degenerated and disappeared, created a similar need for XR to be dosage compensated. Finally, the neo-Y in *D. miranda *is partially degenerated and, therefore, the X_2 _chromosome is also in part dosage compensated. All these phenomena occur by the action of a dosage compensation complex (or compensasome), which recognizes and binds all the chromosomes of drosophilids that require to be compensated [23]. The enrichment of repeats found in the works indicated above [11-13], perfectly correlated with the need for dosage compensation.

In this study, we use the information currently available for multiple *Drosophila *genomes to pursue the research initiated twenty years ago. We were interested in three questions. First, are there species-specific patterns of simple DNA repeats in drosophilids?. Second, is the X chromosome of drosophilids characterized by particular sequence patterns, different from those found in the autosomes?. Third, if indeed the X chromosome has peculiar patterns, which are the forces that generate them?. Here, we show that the characterization in an evolutionary context of oligonucleotide profiles (counts of overlapping words of a given size) offers insights on the forces that shape the sequences of whole chromosomes. Particularly, by comparing oligonucleotide profiles of several drosophilid species, we have obtained a precise view of how chromosomes diversify in the *Drosophila *genus.

## Results

### X chromosome and autosomes of *D. melanogaster *have different compositions

**Figure 1:**
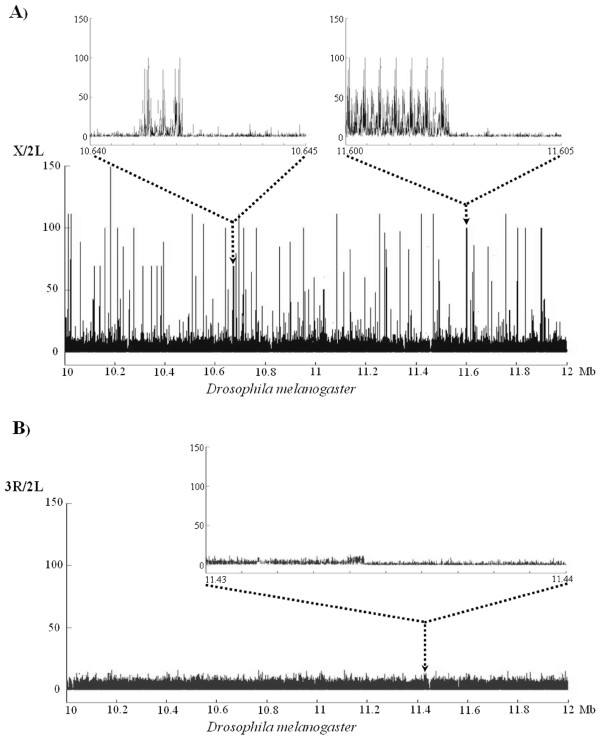
Oligonucleotide profiles (word size *k *= 13) detailing the relative word frequencies between the X and 2L chromosomes (A) or the 3R and 2L chromosomes (B) of *D. melanogaster*. Results for 2 Mb of the X or the 3R chromosomes (x-axis) are shown. The y-axis reflects the relative frequency of words in the two chromosomes after size correction. Details in panel A show the repetitious internal structure characteristic of an X-specific satellite.

The program UVWORD, which determines the types and frequencies of overlapping words of DNA in any given sequence, was used to generate oligonucleotide profiles of the sequences described in Table 1 (see Methods). We logically used *D. melanogaster *as a starting point for our research. First, the general profile of sequence similarity between the X chromosome and the autosomes of this species was established. In Figure 1A, we show a typical result for a 2 Mb fragment of the X chromosome. This figure summarizes corrected X/2L ratios, that is, the number of times that a sequence of the X chromosome (*target*) is found along the X (*source 1*) divided by the number of times that the same sequence is found on the 2L chromosomal arm (*source 2*) and corrected by the relative sizes of those sequences (See the "Chromosomal comparisons" section of Methods for the details). These corrected X/2L ratios should be about 1 for chromosomes for identical composition. However, we observed a very complex landscape, ratios with values up to 150, indicating regions that contain sequences that are highly repeated on the X chromosome but virtually absent on the autosome. When we analyzed several of the broadest peaks, we found them to be formed by tandem repeats (Figure 1A, details). BLAST analyses of these sequences demonstrated that they have high similarity to the X chromosome-specific satellite described by Waring and Pollack [9] and DiBartolomeis et al. [10] (Accession number: X62939). When we compared two autosomes, a qualitatively different picture was obtained. In Figure 1B, ratios obtained in the comparison between chromosomal arms 3R and 2L are detailed. Results were identical in all the other autosomal comparisons. Peaks as those observed in the X/2L comparison were never detected. When any region characterized by somewhat higher ratios was observed in more detail (Figure 1B, top), we simply detected slight departures of the background values. Therefore, we concluded that, in *D. melanogaster*, the profiles of different autosomes are very similar, and totally different from that of the X chromosome.

**Table 1:** General information of the sequences analyzed in this study.

Species	Data repository and release	Muller element A	Muller element B	Muller element C	Muller element D	Muller element E
*D. melanogaster*	NCBI, release 3.1	X (21.8)	2L (22.1)	2R (20.3)	3L (23.3)	3R(27.9)
*D. simulans*	UCSC genome browser, April 2005	X (14.4)	2L (20.7)	2R (18.2)	3L (21.2)	3R (26.0)
*D. yakuba*	UCSC genome browser, November 2005	X (21.5)	2L (22.2)	2R (21.0)	3L (23.9)	3R (28.6)
*D. ananassae*	UCSC genome browser, August 2005	X (12.4)	3R (16.1)	3L (19.7)	2R (23.0)	2L (24.5)
*D. pseudoobscura*	UCSC genome browser, November 2004, freeze 2	XL (23.5)	4 (26.0)	3 (19.0)	XR (23.6)	2 (29.7)
*D. virilis*	UCSC genome browser, August 2005	X (18.9)	4 (19.9)	5 (20.4)	3 (25.0)	2 (19.0)
*D. grimshawi*	UCSC genome browser, August 2005	X (21.0)	3 (14.9)	2 (18.8)	5 (10.7)	4 (24.2)

For each species, the names of the chromosomes and the amount of DNA analyzed per chromosome (in Mb) are indicated. Accession numbers for *D. melanogaster *chromosomes: Chromosome X: NC_004354.1; Chromosome 2L: NT_033779.2; Chromosome 2R: NT_033778.1; Chromosome 3L: NT_037436.1; Chromosome 3R: NT_033777.1. Scaffolds used for *D. grimshawi *were as follows: chromosome X (24821, 25009, 25041); chromosome 3 (24946, 24992, 25029, 25033); chromosome 2 (24999, 25044); chromosome 5 (24997, 25023, 25050); chromosome 2 (24940, 25013).

**Figure 2:**
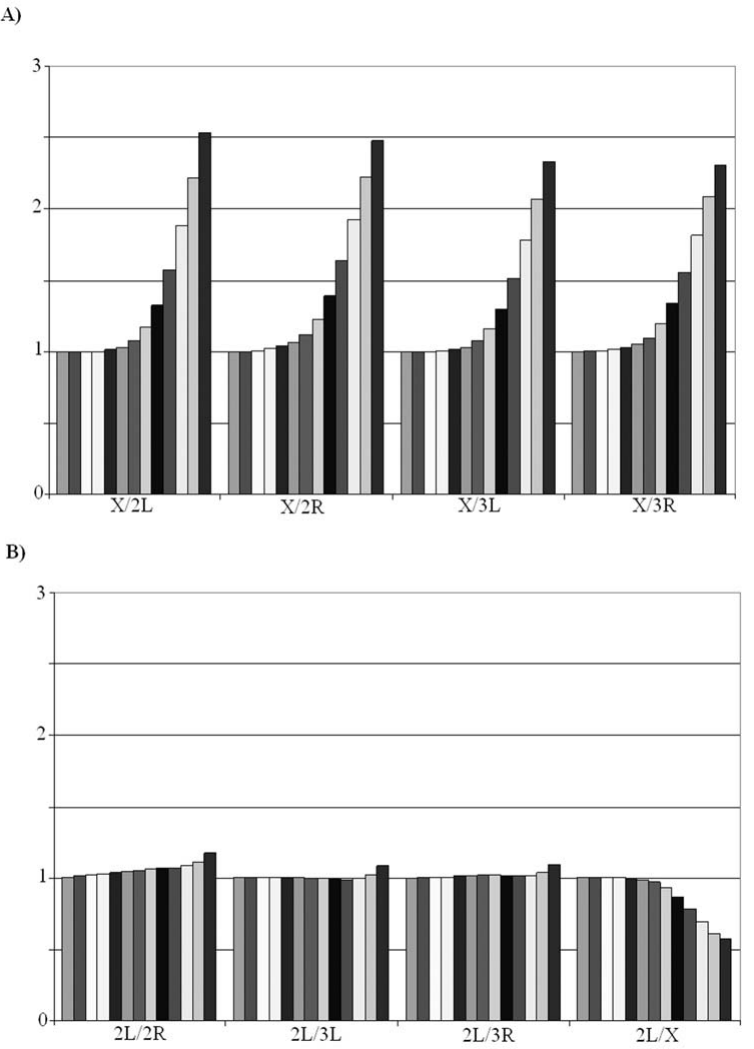
Average values for relative word frequencies in comparisons among *D. melanogaster *chromosomes. To draw panel A, the frequency of all words present in the X chromosome was establish in both the X and autosomes. The histograms show, in the y-axis, the values of the X/autosome ratio, once corrected for chromosome sizes. Values from *k *= 1 to *k *= 13 are depicted from left to right for each comparison. Panel B is similar, but the words were obtained from the 2L chromosomal arm.

We then performed a more precise comparison by determining the average values of the ratios between two *D. melanogaster *chromosomes for different *k *sizes. Results are shown in Figure 2. Figure 2A shows the results for comparisons involving the X chromosome (target) when the two sources are this same chromosome and, in turn, each one of the major autosomal arms. Interestingly, for *k *≤ 6 (small words from mono- to hexanucleotides), the ratios are essentially equal to 1, that is, there is no obvious differentiation among the chromosomes. However, for *k *> 7, the values start to grow, increasing up to about 2.3 – 2.5 when *k *= 13. This indicates that, when the size of the words extracted from the X chromosome is sufficiently large, these words are in average overrepresented on the X respect to the autosomes. However, this could be due to two different causes. First, the X chromosome could have a qualitatively different composition than the autosomes. Alternatively, the effect could be simply due to long word size: it is obvious that, for words obtained from a particular chromosome, the likelihood of them being present in a different chromosome diminishes with word size. Therefore, the results shown in Figure 2A could be caused by the effect of word size in comparisons of chromosomes not significantly different in composition. That this is not a tenable explanation is demonstrated by comparing Figure 2A with the three first panels of Figure 2B, in which different autosomes are compared. Indeed, there is some degree of specificity provided by the words being selected from one of the autosomes (the 2L chromosomal arm) and specificity tends to slightly increase with word size. However, it never reaches values above 1.5. These results thus confirm that the X chromosome contains sequences that are scarce on the autosomes, while all autosomes have very similar compositions.

**Figure 3:**
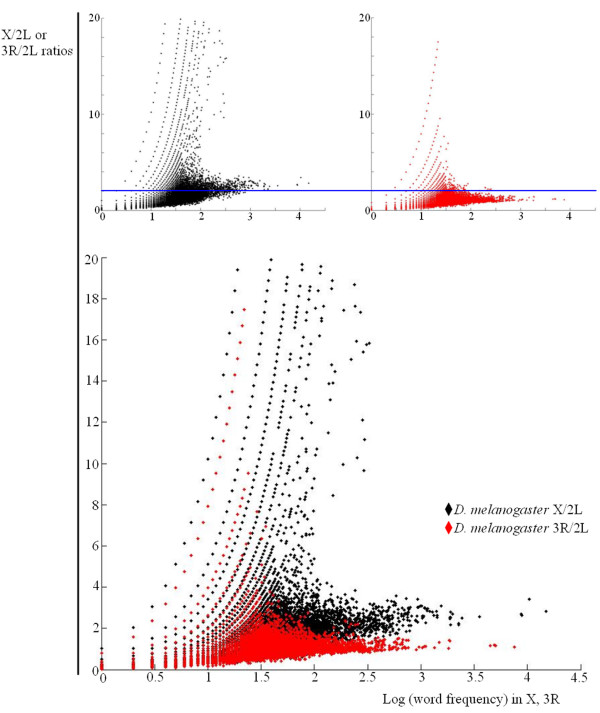
Relative X/2L and 3R/2L values for 5 10^5 ^words randomly obtained from X (black) or 3R (red). For simplicity, ratios above 20 are not shown. The blue line indicate a value of 2.

The last comparison in Figure 2B (2L/X) provides significant additional information. In this comparison, the sequences are obtained from the 2L chromosome, but it turns out that they are proportionally more frequent on the X chromosome that on 2L itself, an effect that increases with word size. This means that the X chromosome not only includes X-specific sequences that are rare or even absent on the autosomes, such as the X-specific satellites that we detected before. It must also contain a second type of sequences, which are present on the autosomes but still are proportionally more frequent on the X. The sequences that explain this last peculiar result can be deduced from data summarized in Figure 3. To generate this figure, sets of 5 10^5 ^words of size *k *= 13 were randomly chosen from either the X chromosome or the 3R autosomal arm. Then, the relative frequencies of those words on the X or 3R respect to a different autosomal arm, 2L, were determined and plotted. On the top, we show the results for each comparison independently. Two differences can be observed. First, very high ratios for sequences found a limited number of times (up to 200–300) are much more frequent on the X/2L comparison. These words correspond to the X-specific satellites. The second difference is less obvious. On the top panels, a line indicating a ratio of 2 has been traced to make clear that many X/2L values for highly repeated words (some present more than 10000 times) are above that line. On the contrary, ratios higher than 2 for highly repeated words are almost absent in the 3R/2L comparison. The superposition of the two figures (bottom panel) allows for these qualitative differences to be more easily observed. This result demonstrates that, apart from the X-specific satellites, there are many different, highly repeated words that are 2 – 4 times more frequent on the X than on the autosomes. In Additional file 1 (first column, *D. melanogaster *data), we have included the 50 most frequent words found on the X, their frequency in the X and an autosomal arm and their X/Autosome (X/A) ratios. These results demonstrate that the highly frequent, X-enriched words are simple repeats. We thus conclude that the *D. melanogaster *X chromosome contains more simple repeats (i. e. it is quantitatively less complex) than the autosomes of this species, in good agreement with the indirect, partial results obtained previously by other authors (see Background section). This quantitatively more repetitious sequence easily explains the results of the right panel of Figure 2B: even when the sequences derive from the 2L autosomal arm, many of them, mostly simple DNA repeats, are more abundant on the X, decreasing the 2L/X ratio. This effect becomes more important with increases in the word size *k *due to the correlation between having more simple DNA and having more long simple DNA-based words.

### X and autosomes show also differences in other *Drosophila *species

**Figure 4:**
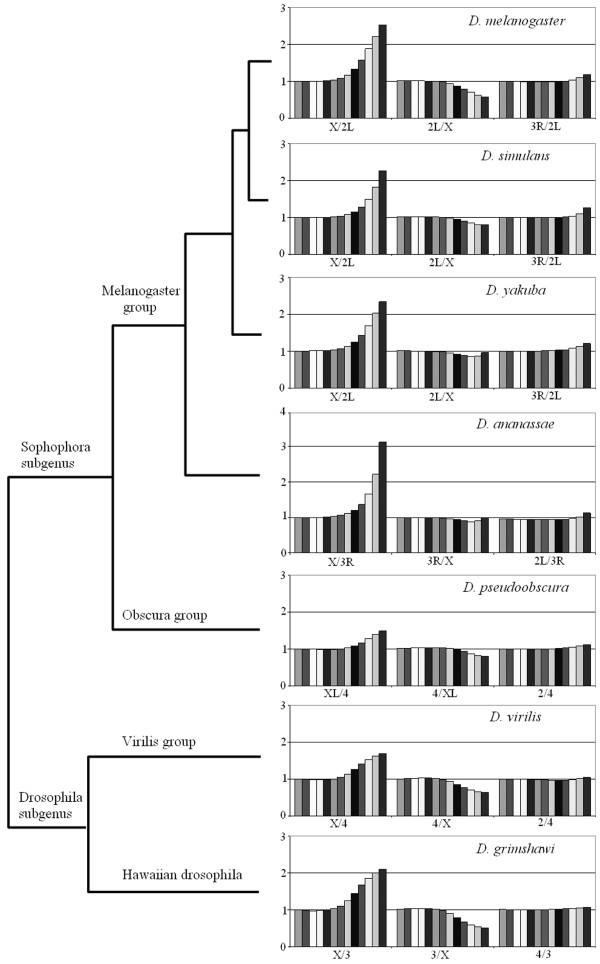
Average values for relative word frequencies in chromosomes of seven *Drosophila *species. As in Figure 2, values from *k *= 1 to *k *= 13 are shown from left to right for each comparison between chromosomes.

**Figure 5:**
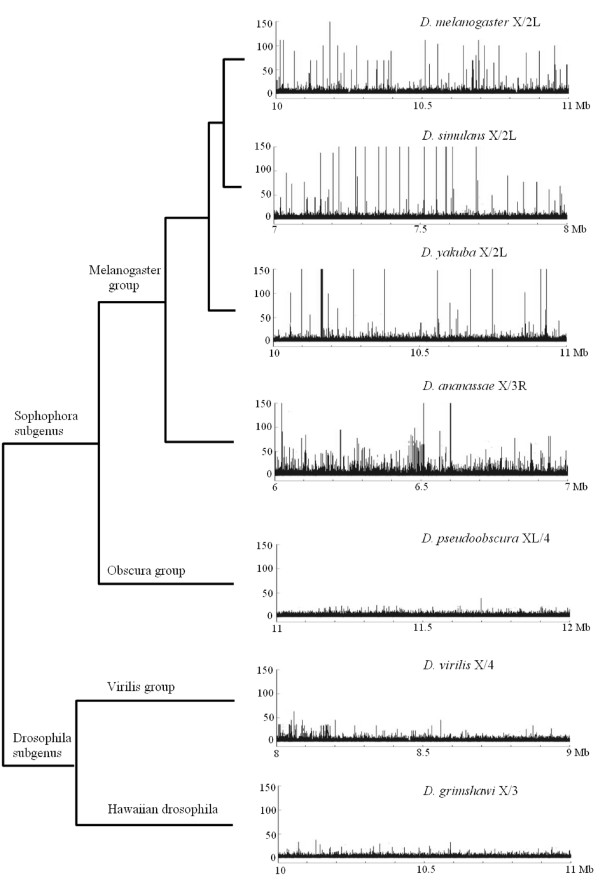
Typical X/autosome profiles for 1 Mb of chromosome X sequences in the seven drosophilid species. Again, the y-axis reflects the relative frequency of the words in the pairs of chromosomes.

The results presented in the previous section demonstrate that oligonucleotide profiling may easily pinpoint global differences in nucleotide composition among eukaryotic chromosomes. Now the question we wanted to tackle was whether the results found for *D. melanogaster *were a peculiarity of that species or the same patterns were also present in other drosophilids. We therefore performed similar analyses in other six species, covering all the phylogenetic range of the *Drosophila *genus. Results are shown in Figures 4 and 5 and in Additional file 1. In Figure 4, we show for each species three typical results in which comparisons of the type X/A, A/X and A_1_/A_2_, where A is an autosome, are summarized for a set of homologous chromosomes. Three conclusions can be drawn from these results. First, the high specificity of the X chromosome is a general feature of drosophilid genomes. Second, the similarity among autosomes that we observed in *D. melanogaster *is confirmed in the rest of species. Third, subtle differences among species exist. For example, the X/A ratios are particularly high in species of the melanogaster group, whereas *D. pseudoobscura *is the species with the least X chromosome specificity. In Figure 5, we show a general profile of the X/A ratios for 1 Mb of X chromosomes of these species. The presence of multiple, very high peaks, corresponding to dispersed X-specific satellites, occurs only in melanogaster group species. We detected localized X-specific repeats in *D. pseudoobscura *and did not find any X-specific satellite in *D. virilis *or *D. grimshawi *(data not shown).

As in *D. melanogaster*, the presence of X-specific satellites contributes but however does not fully explain the results for average X/A ratios described in Figure 4. Comparing the results of Additional file 1 (all species) with those shown in Figure 4, it emerges a clear correlation between the X:A ratio values for the most abundant words, often simple DNA repeats, and the global results described in that figure. To demonstrate that it is indeed a difference in the general amount of simple DNA repeats what mostly determines the elevated X/A ratios and also to characterize whether different species have different types of simple DNA sequences, we established the relative frequency of loci containing strings of mononucleotides, dinucleotides and the most common trinucleotides detected in the seven species (Table 2; here only independent loci were counted using UVCOUNT, see Methods). Three main results became evident. First, we confirmed that there is a strong correlation between the relative frequency of the most common oligonucleotides and the general X/A ratio. For example, this explains the difference detected between *D. melanogaster *and *D. pseudoobscura *(Figure 4): the first species has many more simple repeats on the X than on the autosomes and that difference is much smaller in the second species. The second main result is that, although the most frequent sequences are quite similar among species, there are some species-specific differences. For example, while in *D. virilis *most repeats, and especially (AC/GT)_n_, (AG/CT)_n _and (CG/GC)_n_, are more abundant than in *D. melanogaster*, the opposite is true for the (C/G)_n _repeat (Table 2). A final significant finding is that two species have very abundant, complex X-specific sequences (in bold in Additional file 1). Thus, we have detected that 26 of the most abundant words in *D. virilis *can be assembled to build a 36 bp sequence (GGAGTTATGTTTTGGAACGTCATATCTCCGCGC). This sequence was first discovered as part of the putative mobile element pDv [24]. Similarly, in *D. ananassae*, 25 of the 50 most abundant words in the X do not correspond to simple sequences. Assembling several of those words, a sequence of 38 bp can be built: AAATTTCATAAGGATCGGCCGACTATATCCTATAGCTG. Many complete or partial copies of this sequence, often with some mismatches, are detected in BLAST searches against *D. ananassae *chromosomes. To our knowledge, this sequence had not been hitherto described. The rest of the words fit into a 8 bp repeat (CTGTCCGT) or a 13 bp repeat (TATACCCTTGCAG). The impact of these complex repeats in the genomes of *D. ananassae *and *D. virilis *is significant. In the particular case of *D. ananassae*, the fact that the words derived from the 38 bp sequence are typically 8–10 times more abundant on the X that on the autosomes (see Additional file 1) contributes to explain why this species has the highest ratios in the X/A comparisons of all species examined (Figure 4).

**Table 2:** Percentage of euchromatin that corresponds to the most common mono-, di- and trinucleotides in the X chromosomes and an autosome for the seven *Drosophila *species.

	*D. mel *X	*D. mel *2L	*D. sim *X	*D. sim *2L	*D. yak *X	*D. yak *2L	*D. ana *X	*D. ana *3R	*D. pse *XL	*D. pse *XR	*D. pse *4	*D. vir *X	*D. vir *4	*D. gri *X	*D. gri *3
**A/T**	2.10	1.56	1.85	1.42	1.84	1.36	2.65	2.00	1.42	1.43	1.50	1.91	1.92	1.97	1.94
**C/G**	0.25	0.14	0.22	0.13	0.25	0.13	0.27	0.17	0.35	0.50	0.42	0.15	0.12	0.13	0.10
**AC/GT**	0.78	0.54	0.74	0.54	0.85	0.53	0.58	0.50	0.94	0.89	0.74	1.47	1.12	1.72	1.23
**CA/TG**	0.92	0.67	0.88	0.67	0.99	0.66	0.65	0.59	1.10	1.07	0.90	1.65	1.29	1.90	1.41
**AG/CT**	0.34	0.26	0.33	0.25	0.35	0.26	0.39	0.30	0.91	0.96	0.82	0.66	0.51	0.88	0.67
**GA/TC**	0.31	0.24	0.30	0.24	0.33	0.25	0.39	0.30	0.85	0.90	0.77	0.61	0.47	0.85	0.63
**AT/AT**	0.78	0.58	0.73	0.56	0.70	0.51	0.68	0.47	0.55	0.46	0.53	1.20	1.00	1.56	1.19
**TA/TA**	0.67	0.48	0.63	0.47	0.62	0.44	0.65	0.39	0.45	0.37	0.42	1.10	0.92	1.43	1.06
**CG/CG**	0.05	0.03	0.05	0.04	0.05	0.03	0.03	0.02	0.06	0.05	0.04	0.17	0.10	0.04	0.03
**GC/GC**	0.07	0.05	0.07	0.05	0.07	0.05	0.05	0.04	0.07	0.07	0.06	0.13	0.12	0.08	0.06
**CAA/TTG**	0.87	0.79	0.82	0.77	0.87	0.75	0.73	0.79	0.84	0.89	0.84	1.41	1.31	1.65	1.41
**CAG/CTG**	0.72	0.62	0.71	0.63	0.80	0.67	0.59	0.58	1.01	1.07	0.85	0.98	0.87	1.11	0.88
**TAA/TTA**	0.77	0.82	0.76	0.81	0.75	0.78	0.92	0.82	0.51	0.51	0.64	0.87	0.95	0.96	1.18

**TOTAL**	**6.65**	**5.34**	**6.21**	**5.17**	**6.45**	**5.04**	**6.84**	**5.66**	**6.59**	**6.78**	**6.37**	**8.82**	**7.90**	**10.04**	**8.62**

The total values correspond to the sum of the values for A/T, C/G, AC/GT, GA/TC, AT/AT, CG/CG, CAA/TTG, CAG/CTG and TAA/TTA. In this way, we avoided counting twice sequences that are related.

### Interspecific comparisons

**Figure 6:**
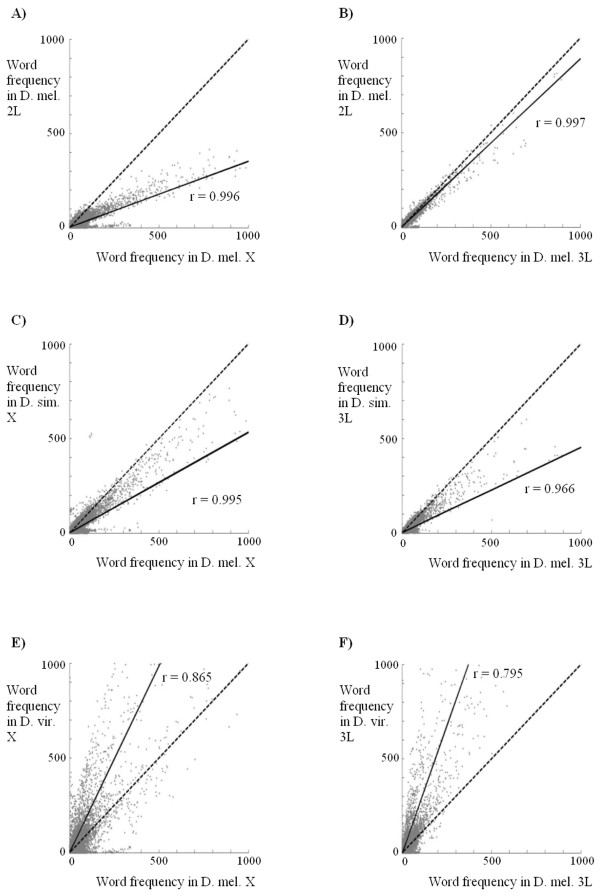
Comparison of the frequencies of 5 10^5 ^words randomly taken from the chromosomes shown in the x-axis in both the chromosome from which the sequences were obtained (x-axis values) and a second chromosome (y-axis values). Panels A) and B) show intraspecific comparisons for *D. melanogaster *chromosomes. Panels C) and D) show comparisons between *D. melanogaster *and *D. simulans *chromosomes. Panels E) and F) show comparisons between *D. melanogaster *and *D. virilis *chromosomes.

If indeed each species has particular amounts and types of simple repetitive sequences, this should leave a general imprint on the chromosomes detectable by interspecific comparisons. The panels in Figure 6 show some intra- and interspecific comparisons. A total of 5 10^5 ^words (*k *= 13) randomly derived from the chromosome specified on the x-axis were counted in both that chromosome and a second chromosome. In the panels, we have plotted those frequencies, once corrected for the relative sizes of the chromosomes. If both chromosomes have identical sequences, they should generate the dashed lines shown in Figure 6, with equal x and y values. If the regression lines traced with the frequencies of both chromosomes (continuous lines in Figure 6) are below that expected line, it means that the sequences are relatively more abundant on the chromosome plotted in the x-axis than in the chromosome plotted in the y-axis, while the opposite is true for regression lines above the dashed line. In addition, correlation coefficients (r) provide an estimation of how related are the compositions of both chromosomes.

Figures 6A and 6B confirm the intraspecific results for *D. melanogaster *obtained above: the X chromosome has more repeats than the autosomes while two autosomes are almost identical. Moreover, the correlation coefficient values close to +1 indicate that the same sequences are present in both X chromosomes and autosomes. Figures 6C and 6D compare homologous chromosomes of two closely related species, *D. melanogaster *and *D. simulans*. It is obvious that *D. melanogaster *chromosomes have more repeated sequences than *D. simulans *chromosomes, in agreement with previous results (summarized in [5]; see also our Table 2 and Additional file 1). Interestingly, comparisons of Figures 6A, 6C and 6D shows that the differentiation between the chromosomes of *D. melanogaster *and *D. simulans *is quite similar to the differentiation between the X chromosome and the autosomes of *D. melanogaster*. This result means that a substantial degree of differentiation among chromosomes can be generated in a relatively short time, because these two species have diverged for only about 5 millions of years [25]. Moreover, the r ≈ 1 values in the analyses shown in Figures 6C and 6D indicate that the words detected are still essentially identical in the chromosomes of these two species. Finally, in Figures 6E and 6F, homologous chromosomes of *D. melanogaster *and *D. virilis*, two species whose lineages split about 63 millions of years ago [25], are compared. Two results are noteworthy. First, *D. virilis *has much more repeated sequences than *D. melanogaster *in both their X chromosome and autosome. Second, composition of the chromosomes is, for these two species, somewhat different. The r values are quite smaller than those obtained intraspecifically. These results thus globally confirm the differences described in Table 2.

We already commented in the Background section of this work that there is evidence for species of the Drosophila subgenus having more simple DNA repeats than those of the Sophophora subgenus. Results summarized in Figures 6E and 6F and in Table 2 are compatible with this idea. However, a more effective way to characterize whether this is the case is to obtain global relative ratios for homologous chromosomes in different species. As an example, Table 3 shows the X/X ratios for different species, that is, the average values of the frequencies of words of size *k *= 13 found in the X chromosome of one species (indicated with the name in the rows) divided by the average values of the frequencies of those same words in the X chromosome of a second species (names indicated in the columns), once corrected by the respective sizes of both chromosomes. Values above 1.00 indicate that the words are more frequent in the species that gives name to the row, while values below 1.00 indicate that the words are more frequent in the species indicated in the column. Results shown in Table 3 confirm those observed in Figures 6C and 6D. They also extend the results to demonstrate that the two species of the Drosophila subgenus (*D. virilis *and *D. grimshawi*) contain qualitatively more repeats than the five species of the Sophophora subgenus analyzed. On the other hand, the results within subgenera are more complex. Most significantly, in seven cases the two reciprocal comparisons between two species are both above 1, a result that can only be explained by both species having similar amounts of repeats, but those repeats being, at least in part, species-specific.

**Table 3:** X/X ratio values among *Drosophila *species (*k *= 13).

	*D. mel*. X	*D. sim*. X	*D. yak*. X	*D. ana*. X.	*D. pse*. XL	*D. vir*. X	*D. gri*. X
*D. mel*. X	--	1.71	1.54	2.06	1.65	**0.50**	**0.42**
*D. sim*. X	0.50	--	0.99	1.32	1.08	**0.35**	**0.29**
*D. yak*. X	0.89	1.95	--	1.55	1.08	**0.38**	**0.31**
*D. ana*. X	1.54	3.34	2.00	--	2.02	**0.72**	**0.63**
*D. pse*. XL	1.34	2.95	1.51	2.19	--	**0.45**	**0.35**
*D. vir*. X	**3.24**	**7.74**	**4.19**	**6.27**	**3.57**	--	0.89
*D. gri*. X	**4.26**	**10.03**	**5.39**	**8.53**	**4.42**	1.40	--

In bold, comparisons between species of different subgenera.

### Evolution of the neo-X chromosomal arm of *D. pseudoobscura*

**Figure 7:**
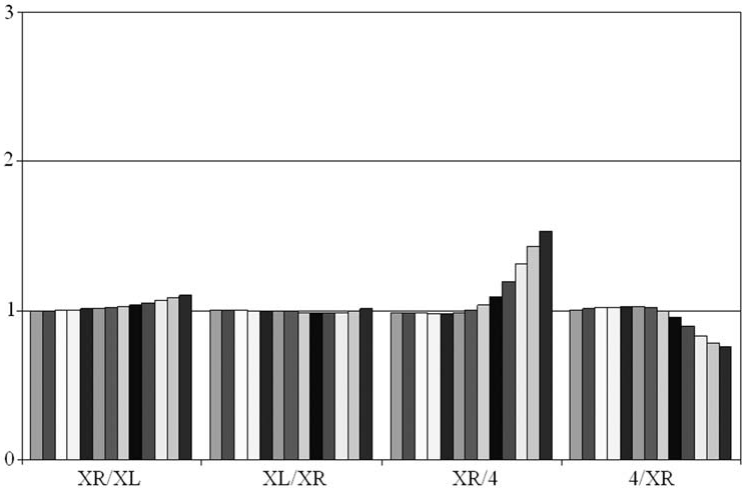
Average values for relative word frequencies in comparisons involving the XR chromosomal arm of *D. pseudoobscura*. Again, values from *k *= 1 to *k *= 13 are shown for each comparison.

**Figure 8:**
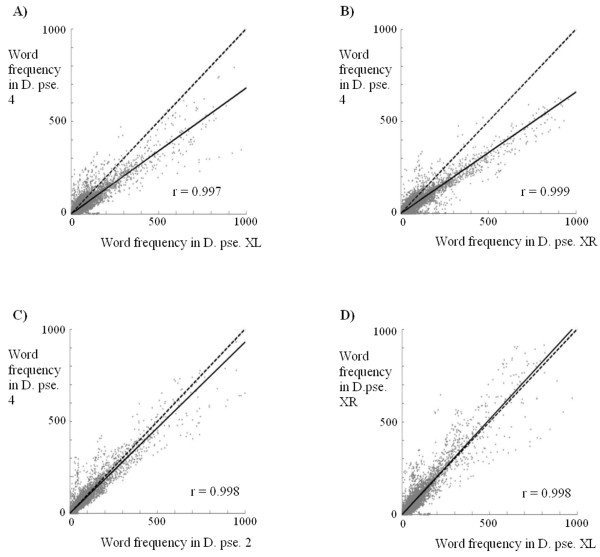
Comparisons of frequencies for 5 10^5 ^words obtained from different *D. pseudoobscura *chromosomes. Details as in Figure 6.

In *D. pseudoobscura*, Muller's element D, which is normally autosomic (e. g. in *D. melanogaster*, it corresponds to the 3L arm), suffered a translocation that converted it into a new X chromosome arm, called XR. This translocation has been estimated to occur more than 10 but less than 18 millions of years ago [25,26]. The non-translocated homologous chromosome degenerated and probably became the current Y chromosome of this species [27]. The availability of essentially complete genomic data for *D. pseudoobscura *allows to characterize whether chromosomal profile changes have occurred in association with this conversion of an autosome into an X chromosomal arm. Figures 7 and 8 show the results for comparisons of the XR chromosomal arm with the XL arm or with an autosome. As it can be seen in Figure 7, left panel, the general comparison for XR and XL arms indicates that they are virtually identical. Inspection of Figures 4 and 7 allows to see that the results for comparisons of XR or XL with an autosome are also identical. In Figure 8, we again show the results for 5 10^5 ^random words, this time obtained from different *D. pseudoobscura *chromosomes. Figures 8A and 8B show that XL and XR provide essentially indistinguishable results when compared with an autosome. Figures 8C shows that, as happens in other species, *D. pseudoobscura *autosomes have the same composition. More interesting is Figure 8D in which we show that the XL and XR chromosomal arms contain patterns of words that are also indistinguishable, with a correlation coefficient which is about 1.0. These global results are also confirmed by data shown in Table 2 and Additional file 1. These are striking results, because they demonstrate not only that the neo-X (XR) arm has greatly diverged from the autosomes but also that it has *converged *to the XL arm. We can conclude that both X chromosome arms have, after 10–18 millions of years of being together, a peculiar and essentially identical sequence pattern.

## Discussion

We have shown that the X chromosomes and the autosomes of *Drosophila *species have different global compositions. The X chromosome is enriched in simple repeats and also contains, in some species, complex X chromosome-specific sequences. On the other hand, all autosomes within a *Drosophila *species have identical compositions. There is however variation among species, with those in the melanogaster group being the ones with the highest level of global X chromosome specificity and *D. pseudoobscura *the one with the lowest (Figure 4). The enrichment of simple DNA sequences on the X chromosome is not accompanied by a general modification of the types of repeats. In all species tested, the words found on the X and on the autosomes are the same, only their frequencies change. This is shown by the high correlations observed for comparisons between X and autosomes (Figures 6, 8). A significant difference in chromosome composition is detected for comparisons between Drosophila subgenus and Sophophora subgenus species. Sophophorans contain less simple repeated DNA (Tables 2, 3; Additional file 1). This result correlate with the fact that the two species of the Drosophila subgenus have larger euchromatic genomes (about 140 Mb) than the rest (about 120 Mb; data from [28] at the DroSpeGe web page. See [29]). In fact, simple DNA repeats could explain a substantial part of the differences in genome size. The repeats detailed in Table 2 (a fraction of the total present in those chromosomes) account for about 6 Mb of the 117 Mb euchromatic genome of *D. simulans *and for about 12 Mb of the 140 Mb euchromatic genome of *D. grimshawi*. This means that they alone explain about 26% (6/23) of the difference of euchromatic genome size between these two species. Even after all these results, before concluding that this difference between subgenera is general we must consider that the number of species examined is still small. In fact, indirect results suggest that some species may not fit this pattern. For example *D. subobscura*, a sophophoran species quite closely related to *D. pseudoobscura*, seems to have many repeats [30]. In addition, there is evidence for significant differences among closely related species of the Drosophila subgenus [31].

The finding that *D. melanogaster *has many (AC/TG)_n_, (AT/AT)_n_,(AG/CT)_n _and (A/T)_n _repeats was previously described by other authors [13,18,32,33]. We have detected also a considerable proportion of (AAC/GTT)_n_, (AGC/GCT)_n _and (AAT/ATT)_n _simple sequences in this species. These are also the predominant repeats in the rest of melanogaster group species. In addition, all the species of this group have considerable amounts of X chromosome-specific satellites, which are especially frequent in *D. ananassae *(see Additional file 1). In general, the most abundant simple repeats in melanogaster group species are also the most abundant in the rest of drosophilids. However, there are some differences. For example, melanogaster group species contain less (AG/CT)_n _repeats than the rest (Table 2). These subtle differences, together in some cases with the appearance of species-specific sequences (*D. virilis, D. ananassae*), lead to global changes when homologous chromosomes of distantly related species are compared (e. g. Figure 6; *D. melanogaster *– *D. virilis *comparisons). Modifications of the sequences of these chromosomes occur at a relatively rapid timescale (see also Figure 6; *D. melanogaster *– *D. simulans *comparisons). A final significant result is that we have observed convergence in the global sequence pattern between the XL and XR arms of *D. pseudoobscura*. The conversion of XR from an autosome into a neo-X chromosomal arm has led to an increment in its amount of simple DNA repeats up to levels which are identical to those in XL, the original arm of the X chromosome (Figures 7 and 8). This process, occurred in the last 10 to 18 millions of years, suggests that the X chromosome in a given species has an optimal composition, different from the autosomes, to which neo-X chromosomes tend.

We may now ask which are the forces that are behind all these patterns. In our opinion, the systematic finding of X-chromosome specificity in all species, the almost identical results for autosomes within a species, the correlation between evolutionary relatedness and differences in the simple DNA components and the finding of convergence between the XL and XR chromosomal arms rule out that these changes are due to chance alone. Systematic forces must be operating that contribute to X chromosome differentiation from the autosomes and to autosome homogeneity within a species. Related forces must explain why different species have different amounts and types of simple DNA repeats. Understanding the causes of these patterns may be of broad interest, because similar trends are present in other animal species. For example, results in primates are consistent with a rapid modification of repeat content in closely related species and differences in simple repeats between the X chromosome and the autosomes have been also observed [34-37].

A first interesting question is how to explain differences between closely related species such as the ones that we have studied. This is a classical problem. Several authors have recently discussed the reasons why related organisms have different amounts of non-coding and/or repetitive DNA, often in the context of the impact that those changes may cause on genome size [38-45]. The summary is that there are many forces that influence the global composition of genomes. Some of them imply differences in mutation rates while others are related to changes in selective regimes. The problem is that the particular contributions of those forces are unknown. For example, genome size tends to correlate with increases in simple DNA and microsatellites [40,42,46], increases in intron size (e. g. ref. [47]), increases in transposable element number [41], etc, while a high rate of nucleotide deletion tends to correlate also with a small genome size [48-50]. However, none of these forces fully determines genome size, which leads to multilevel hypotheses involving many different parameters (see discussions in [38,43,44,51]).

We can rule out some possible explanations for the observed interspecific differences in the amounts of simple DNA sequences. For example, changes do not seem to correlate with external features, such as the geographical distribution of these species, which might be associated to differences in life history traits. *D. simulans *and *D. virilis*, which are in the extremes of the distribution of simple DNA content, are both human commensals and thus widely distributed [52]. Also, a weak correlate can be obtained between size of the organisms and simple DNA content (and genome size), because *D. virilis *and, especially, *D. grimshawi *individuals are bigger than the sophophoran species considered. However, organism size obviously does not explain the differences observed for *D. melanogaster *and *D. simulans*, two sibling species.

Agreement with internalist hypotheses is also difficult. Some simple explanations can be probably dismissed. For example, the rate of DNA deletion does not correlates well with simple DNA content, because *D. melanogaster *and *D. virilis *have similar rates [48]. The rate of point mutations in unconstrained regions seems to be also similar in *D. virilis *and melanogaster group species [53]. It is unclear whether recombination contributes to explain the patterns observed. On one hand, increased recombination may decrease genome complexity if recombination favors the generation of repeats. On the other hand, selective pressure to increase simple DNA repeats, for them to act as recombinogenic sequences, could occur in some species (see Refs. [42,54-58]). In either case, we would expect a strong correlation between recombination rates and amounts of simple DNA repeats. However, in drosophilids, this correlation is not obvious. First, we would expect a decrease in simple DNA repeats in species with low chromosomal polymorphism, which lack inversions that restrict recombination. However, this pattern is not observed. For example, *D. simulans *is chromosomically monomorphic (reviewed in [59]), but so is *D. virilis *[60]. In addition, the length of the chromosomes in map units, an indication of the likelihood of recombination, correlates only partially with the amount of simple repeats. For example, in reasonable agreement with their respective amounts of simple DNA repeats, the X chromosome of *D. virilis *has 170.5 map units, while the *D. pseudoobscura *XL chromosome has 157.6 units and the *D. simulans *X chromosome only about 66 [61-63]. However, *D. simulans *has total map distances which are about 30% longer than those of *D. melanogaster *[62], while the amount of repeats in all *D. simulans *chromosomes is smaller. In particular, the X chromosome has about the same length in recombination units in those two species [62], in spite of their significant difference in repetitive DNA (Figure 6C). In summary, although differential recombination rates may contribute to generate the patterns observed, they cannot explain them all.

Apart from recombination, there are three internal forces able to increase repeatedness that may also contribute to explain the interspecific differences obtained. First, differential intrinsic slippage rates among species, generating more abundant and larger microsatellites in some of them. Second, species-specific amplification of satellites. Third, an increase in mobile element number in some species. These three forces may positively correlate. We have obtained evidence for a relevant decrease in chromosome complexity in some species to be associated to the amplification of relatively complex, repeated sequences (X-linked satellite in melanogaster group species; complex repeated sequences in *D. ananassae *and *D. virilis*). In the case of the 36 bp sequence detected in *D. virilis*, it has been described as included in a putative mobile element called pDv [24]. It has been suggested that differential amplification of mobile elements may change in a short time the global sequence pattern of a chromosome, especially if the elements have simple internal sequences that contribute to the generation of new microsatellites [64]. So far, evidence for this type of process is not strong, but we have not found any result contradicting this hypothesis, so it deserves further study.

A second pattern that requires explanation is why X and autosomes are so different. This could be due to either mutational or selective forces acting differentially in X chromosomes vs. autosomes. In *Drosophila*, at least in analyses involving closely related species, there is no evidence for strong differences in mutation or selective regimes acting on coding regions of the X chromosome versus those found in the autosomes (reviewed in [65]). In our opinion, this leaves open two possible explanations. The first option is differential recombination rates among chromosomes. Due to the fact that in *Drosophila *males do not recombine, two thirds of the X chromosomes but only two fourths of the autosomes recombine in each generation. Given a positive association between recombination and generation of repeats, this could lead to an increase of repeats on the X chromosomes. We think however that recombination may again contribute but does not fully explains our results. This hypothesis predicts a correlation between relative recombination rates and relative amounts of repeats that it is not observed. Thus, comparing *D. melanogaster*, a species with a high X/A ratio, with *D. pseudoobscura*, the species with the lowest X/A ratio (Table 2), we would expect the former to have higher relative recombination rates in the X respect to the autosomes. However, the opposite if found. For example, the X/2L relative euchromatic recombination rate is 1.22 for *D. melanogaster *(data from Flybase.org) while the same rate for the homologous chromosomes of *D. pseudoobscura *(X/4) is 1.94 [63]. Thus, recombination may influence but does not seem to determine the relative proportion of repeats in X chromosomes and autosomes.

The second posible explanation is that the pattern observed derives from a functional requirement for simple repeats on the X. Our favorite explanation is that it is related to the need of dosage compensate the X chromosome. More precisely, the acquisition of dosage compensation might require a modification of the DNA of a chromosome to make it more repetitious. This could be caused by the dosage compensation complex using simple sequences to recognize the X chromosome. Alternatively, an increase in simple DNA might contribute to increased transcriptional levels by allowing the complex to act on appropriate chromatin domains (see discussion in [22]). The idea that repeats in some way contribute to dosage compensation is old, but always lacked empirical support (e. g. see comment in [66]). Several recent analyses of the dosage compensation complex binding sites do not really confirm or refute this hypothesis, because no obvious consensus sequence required for binding has emerged [67-71]. However, results obtained by Peter Becker's group [68,69,71] suggest that repetitious sequences, rich in CA/TG and GA/TC dinucleotides, may cooperate to facilitate the binding of the complex. If this is the case, X chromosome-specific binding could be achieved by increasing the density of simple DNA repeats on the X respect to the autosomes. Interestingly, a related situation seems to explain the recognition of the X chromosome by the *Caenorhabditis elegans *dosage compensation complex [72,73]. Dosage compensation in humans, associated to X chromosome inactivation, may also be related to the enrichment of repetitive sequences on regions of the X chromosome [74-76].

As a final aside, we must point out that this work shows how useful is to perform oligonucleotide profiling studies of eukaryotic chromosomes using long words (e. g. *k *= 13). The subtle differences that exist among chromosomes or among species can be very simply uncovered by analyses using long, rare, words, while they are difficult to demonstrate when shorter, more unspecific sequences are analyzed. For example, Stenberg *et al*. [77] characterized by multivariate analyses the differences between chromosomes of *D. melanogaster*, *D. simulans *and *D. pseudoobscura*, using short words (up to *k *= 6). With hexamers, they found strong characteristic signatures of the Muller F elements (dot chromosomes) of these species and just a weak differentiation of the X chromosomes of *D. melanogaster *and *D. simulans*, but not *D. pseudoobscura*, respect to their autosomes. With our approach, based on larger words, we have detected clear differences for all three species.

## Conclusion

Oligonucleotide profiling allows for a rapid characterization of the patterns of sequence evolution. We have shown that chromosome profiles are quite similar among *Drosophila *species, with the X chromosome being always simpler than the autosomes. However, the particular sequences that confer this simplicity to the X vary among species. The differences observed among closely related species and the identical profiles of X and neo-X chromosomes suggest that strong forces are acting on relatively short periods of time to generate these patterns. We suggest that the combined effects of differential recombination, differential generation of simple DNA repeats and natural selection caused by the need of dosage compensation may explain our results.

## Methods

### Genomic data

We used genomic data for five species of the Sophophora subgenus and two species of the Drosophila subgenus. Within the Sophophora subgenus, four species of the melanogaster group (*D. melanogaster*, *D. simulans*, *D. yakuba *and *D. ananassae*) and one species from the obscura group (*D. pseudoobscura*) were analyzed. The two Drosophila subgenus species were *D. virilis *(virilis group) and *D. grimshawi *(hawaiian Drosophila). These species were chosen for two reasons. First, to cover all the range of divergence times within the genus, from perhaps 5 millions of years of divergence (*D. melanogaster *– *D. simulans*) to about 63 millions of years (species of the Sophophora subgenus vs. species of the Drosophila subgenus) [25]. Second, because at the time we started our study (beginning 2006) they were, among the eleven ongoing drosophilid genome projects, the ones with the best available sequences. Table 1 describes the sequences used in this study – ordered according to the standard nomenclature of Muller elements, which correspond to homologous chromosomal arms – and their origin. We centered our attention on the X chromosomes and the longest autosomes. The dot chromosomes (Muller F elements) were not considered. All the analyzed sequences were euchromatic. For *D. melanogaster*, *D. simulans *and *D. yakuba*, the chromosomes were already assembled in the databases. For *D. pseudoobscura*, we added together all the pieces of a same chromosome in a single file. Finally, for the other three species we added together several scaffolds that corresponded to regions homologous to *D. melanogaster *chromosomes. Sizes of the final files ranged from 10.7 to 29.7 megabases (Mb). According to the most recent data (December 2006 assembly of the *Drosophila *genomes; see [78] and the DroSpeGe database [28]), these files contained from about 71.1% (*D. grimshawi*) to 97.2% (*D. pseudoobscura*) of the euchromatin of these species, with the average being 85.2%. Therefore, an assuming no extreme biases occurred in the sequencing projects, our samples may be considered fully representative of their euchromatic genomes.

### Oligonucleotide profiling

Characterization of the chromosomal profiles was performed using a program called UVWORD [79]. This program characterizes, using a sliding-window approach, all overlapping oligonucleotides of a particular size *k *present in a particular sequence (*target sequence*) and then establishes their frequency in another sequence (*source sequence*). The user may select a value of *k *such that 1 ≤ *k *≤ 14. If source and target sequences are the same, for example a particular chromosome, the program provides the frequency of all oligonucleotides of the chosen size *k *in that chromosome. If, on the other hand, source and target are two different chromosomes, the program counts how many times each oligonucleotide in the target chromosome is present in the source chromosome. Comparison of the results for two different sources allows for a rapid characterization of the similarity of two DNA sequences (see below: Chromosomal comparisons; [79]). Along this work, we have used values of *k *ranging from 1 to 13 nucleotides. Most of the analyses requiring long words were performed using 13 nucleotides. In general, we preferred *k *= 13 because 13 is a prime number, being thus less affected by the presence of repeats based on dinucleotides, trinucleotides, etc. In all analyses, results for both chains of the DNA molecules were added together. Complex repeats in *D. ananassae *and *D. virilis *were manually assembled from results in Additional file 1.

### Calculation of the number of sites containing simple DNA sequences

Because UVWORD counts overlapping words, a microsatellite may generate adjacent identical sequences that will be counted multiple times (e. g. with *k *= 13, a (CA)_8 _microsatellite will generate two CACACACACACAC and two ACACACACACACA sequences). Therefore, this program cannot count the number of independent sites in which a particular perfect repeat is present along a chromosome. To solve this problem, we generated a second program, called UVCOUNT. This program searches for a given sequence or arbitrary size establishing its frequency and positions in a DNA sequence. After UVCOUNT analyses were completed, results were filtered, in order to count just once the words that overlap. This combined analyses provided the number of independent loci that contained a sequence of interest and their positions. Only strings of six or more nucleotides (i. e. at least six contiguous identical mononucleotides, three contiguous identical dinucleotides or two identical trinucleotides) have been counted in the analyses shown in Table 2. In those analyses, again, results for both chains of a double helix were counted together.

### Chromosomal comparisons

To obtain a global value of similarity for two chromosomes, we first obtained the counts for all oligonucleotides present in the target sequence (one of the two chromosomes) in each of the two source sequences (i. e. each of the two chromosomes in which we were interested, which we called "source 1" and "source 2" above). For each chromosome, the counts were summed and averages were obtained. Then, the averages of both sources were divided one by the other. This final proportion was corrected to account for differences in size between the two sequences. In random sequences of long size, this final corrected value would be about 1.

## Authors' contributions

The three authors contributed to the development of the strategies of analyses implemented in UVWORD and UVCOUNT. Miguel Gallach performed all the analyses shown here and generated the tables and figures of the manuscript. Vicente Arnau wrote and tested the programs. Ignacio Marín devised and coordinated the research and wrote the manuscript. All authors read and approved the final manuscript.

## Supplementary Material

Additional file 1 Additional table with frequent words. Most frequent words found in the X chromosome of the seven species, and their frequencies both for the X and for an autosome. Click here for file
