# Supplementary material for: Global patterns of sequence evolution in Drosophila
Source: BMC Genomics. 2007 Nov 9;8:408. doi: 10.1186/1471-2164-8-408 (PMC2180185; doi:10.1186/1471-2164-8-408)
Supplement: Additional file 1 — Additional table with frequent words. Most frequent words found in the X chromosome of the seven species, and their frequencies both for the X and for an autosome. [file 1471-2164-8-408-S1.doc]

**Additional File 1. Most frequent words found in the X chromosome of the seven species, and their frequencies both for the X and for an autosome. In *D. ananassae* and *D. virilis,* complex DNA sequences are indicated in bold. The summaries (bottom) indicate the number of different words in these lists that either correspond to variations in the indicated simple DNA sequences (in parentheses) or belong to the complex sequences described in the text.**

(A): 25 (AGC): 3 (AG): 2 (A): 17 (AGC): 6 (ACAT): 2

(AT): 7 (AAC): 3 (ACAT): 2 (AC): 10 (ATCT): 4 (ATC): 1

(AC): 4 (AAT): 3 (C): 1 (AT): 8 (AG): 2

**Additional file 1. (continued)**

(A): 19 (AT): 4 (AG): 2 (A): 12 (AC): 2

(AGC): 11 (AAC): 3 (ACAT): 2 (AGC): 3 (AT): 1

(AC): 5 (AAT): 3 (C): 1 (AAC): 3 Complex: 25

**Additional file 1. (continued)**

(ACAT): 13 (AAC): 3 (C): 1 (AGC): 11 (A): 3 (ACAT): 2

(AC): 11 (ACG): 3 (ATC): 1 (AG): 10 (AAC): 3 (AT): 1

(AGC): 10 (A): 2 (AC): 8 (AGG): 3 (C): 1

(AG): 5 (AT): 1 (ACTGCC): 4 (ACG): 3 (ATACAG): 1

**Additional file 1. (continued)**

(AC): 4 (AAT): 3 (AG): 2 (AT): 17 (AAC): 5 (ACAT): 2

(A): 4 (ACAT): 3 (AT): 1 (AC): 11 (A): 4 (AG): 2

(AAC): 3 (AGC): 3 Complex: 27 (AGC): 6 (AAT): 3
